# A dry lab for medical engineers?

**DOI:** 10.1186/1750-1164-3-9

**Published:** 2009-07-31

**Authors:** Francesco Rulli, Angelo Maura, Gabriele Galatà, Giulia Olivi, Michele Grande, Attilio M Farinon

**Affiliations:** 1 Department of Surgery, University of the Study of Rome "Tor Vergata", Rome, Italy; 2 Medical Engineering Course – Innovative Surgical Technologies, University of the Study of Rome "Tor Vergata", Rome, Italy

## Abstract

**Introduction:**

We describe a teaching and training method with objective evaluation to improve medical engineering students' knowledge and analysis skills about Minimally Invasive Surgery (MIS) instrumentation and techniques through hands-on experience. Training has been scheduled during a three-month course.

**Methods:**

Twenty medical engineering students were trained to perform three times on a pelvic trainer a sequence of standardized drills connected with the selected MIS techniques, in order to improve their dexterity. The time required to perform each dexterity drill was recorded in seconds.

Then, the participants were divided into groups and asked to write an essay about an instrument they chose, analyzing and criticizing the instrument itself.

**Results:**

All the trainees showed steady improvement in skill acquisition on the laparoscopic simulator and discussed their essays, making proposals in order to improve the instrument they tested.

**Conclusion:**

Significant improvement in performance with increasing skillness has been measured; during the course and during their discussion the participants showed deep knowledge of the instrument, ability to analyze and criticize it and ability to make improvement proposals.

Dry lab experience for medical engineering students is useful for teaching and improving analysis and management of laparoscopic devices, allowing identification of problems and developing better devices.

## Introduction

The advances in the experience with endoscopic video-assisted techniques have led to the introduction of dry laboratories by several centres. MIS requires training, crediting and caseload, which are not always available in small centres. Moreover, since it is a technologic surgery, new instrumentation need to be tested in a non-clinical environment and eventually discussed with technicians, particularly with bio-medical engineers.

In this field an increase in the use of dry and wet surgical laboratories has been observed. A dry laboratory (dry lab) is specific to work with dry stored materials, electronics and/or large instruments, where, unlike wet laboratories, biological tissues (living or dead) are not utilized. These dry labs have equipped workstations for practicing endoscopic techniques, in a realistic setting, on phantoms and organ models.

These environments are considered essential tools to design a minimally invasive surgery training program, gaining, certifying and improving the expertise. Many institutions, universities, centres of excellence are equipped with dry labs in order to provide a teaching, training and evaluation instrument of the fundamental technical skills for surgeons in a laboratory setting [1].

A dry lab provides laparoscopic simulators such as box trainers and computer-based reality platforms [1]; the objective of the simulator-based curricula is the attainment of basic skills while working in a cheap and pressure-free environment, in order to transfer them from the laboratory to the clinical experience, and to allow the trainees to focus on more complex issues in the operating room.

We describe a teaching and training method with objective evaluation to improve the medical engineering students' knowledge on Minimally Invasive Surgery (MIS) instrumentation and techniques through hands-on experience, in order to allow the students to analyze and criticize the devices they tested. Training has been scheduled during a three months course.

## Methods

The course was kept at the "Tor Vergata" University of Rome in a laboratory setting. It involved 20 medical engineering students without any experience in laparoscopy.

The students were trained during hands-on sessions of the duration of three hours throughout a three-months course. During the experience, the students underwent a theoretical and practical course. The theoretical section consisted of review lectures and discussions about the general background of the procedure, and about selection criteria for the tasks. In addition, an introduction to the instrumentation materials was given. After the theoretical section, the students were allowed to exercise on the box trainer. The laparoscopic simulator consisted of a traditional pelvic trainer.

Hands-on exercises were performed on a simulator in order to assess whether specific training exercises were helpful for medical engineering students to familiarize with surgical devices and techniques.

On the simulator, during the practical section of the course, every student was asked to perform a sequence of standardized drills three times in order to improve their dexterity, deep perception, instrument-targeting accuracy, visual and spatial abilities, and hand-eye coordination; time to complete the task was recorded in seconds.

In more detail, the exercises consisted of: 1) clipping and cutting (a plastic 5 mm diameter tube, marked at its half, was placed in the pelvic trainer; the students had to put a clip before and after the mark and then cut the tube on the mark, so as to simulate clipping and cutting of a vessel); 2) cutting a paper (a square 10 cm × 10 cm paper sheet was placed in the pelvic trainer; the students had to cut it along one of the diagonals, so as to simulate cutting with laparoscopic scissors); 3) positioning a needle (a rubber parallelepiped was placed and fixed in the pelvic trainer; the students had to simulate the positioning of a needle using needle-holders); 4) intra-corporeal knotting (the students had to simulate an intra-corporeal knot on the above rubber parallelepiped); 5) carrying out specimen with EndoBag (the students had to cut a piece of the above rubber parallelepiped and to carry it out using an EndoBag™ (Autosuture™, Norwalk, CT, USA) specimen retrieval system).

After this practical activity, the students were divided into 4 groups and asked to choose an instrument they utilized, to analyze it and to write and discuss a short essay about it, in which they had to describe the instrument (application, purpose, components, functionality, manufacturing techniques and materials) and had to criticize it, pointing out advantages and disadvantages of each instrument and eventually proposing new solutions in order to improve the instrument itself.

The devices chosen by the four groups of students were [2]:

• Roticulator Endo Mini-Shears™ (Autosuture™, Norwalk, CT, USA)

• Blunt tip trocar with balloon tip (Autosuture™, Norwalk, CT, USA)

• DST Series™ GIA™ 60 (Autosuture™, Norwalk, CT, USA)

• EndoClinch™ II (Autosuture™, Norwalk, CT, USA)

### Statistics

Student t test was used to compare the times required to perform the first vs. the second and the last sequence of drills in order to test the efficacy of training course. All data are exposed in terms of mean ± standard error (SEM). A value of p < .05 was considered statistically significant.

## Results

**Figure 1:**
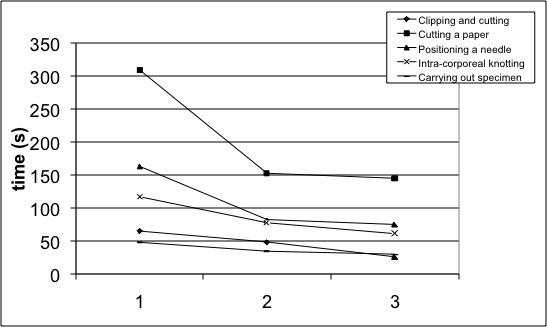
**Time needed to complete the tasks**.

Using the simulator, the overall time required to perform the first time the sequence of drills was higher if compared to one required to perform the second and the third ones (clipping and cutting: 65 ± 27 vs. 48 ± 19 vs. 26 ± 8 sec; cutting a paper: 309 ± 158 vs. 152 ± 59 vs. 145 ± 72 sec; positioning a needle: 163 ± 176 vs. 80 ± 32 vs. 75 ± 37 sec; intra-corporeal knotting: 117 ± 97 vs. 77 ± 50 vs. 61 ± 45 sec; carrying out specimen with EndoBag: 48 ± 26 vs. 33 ± 13 vs. 30 ± 15 sec). The difference was statistically significant between the first and the second time performance for every task (p < .05). Moreover, the second performance required a longer time than the third; the difference was close to significance (p = .05) (fig. 1).

The comparison between the first and the third performance times was statistically significant for every task (p < .05).

The students proposed the following improvements and criticisms about the devices they analyzed:

• Roticulator Endo Mini-Shears™ (Autosuture™, Norwalk, CT, USA)

### Ergonomics

1) The handle causes traumas and local paralysis of fingers.

2) Shaft articulation can be activated only by opposite hand to the hand that holds the device.

### Improvements

**Figure 2:**
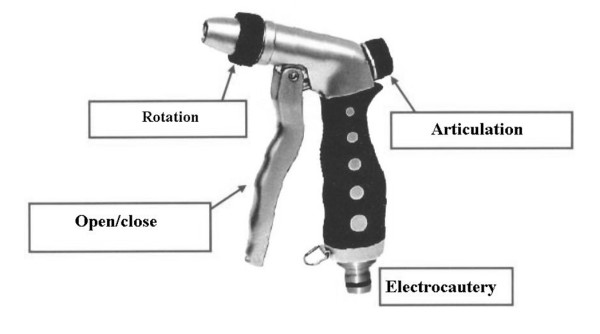
**A more ergonomic handle for the Roticulator Endo Mini-Shears™**.

**Figure 3:**
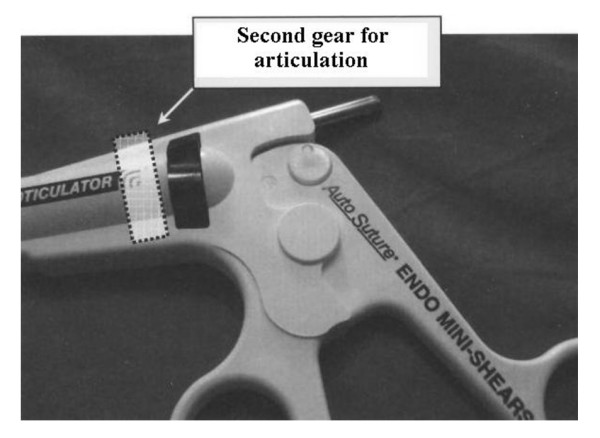
**Proposed position for the second gear on the Roticulator Endo Mini-Shears™**.

1) Building of a more ergonomic handle (fig. 2).

2) Replacement of the grooved collar for articulation with a second scalloped dial located on the handle, to be activated by the same hand that holds the device (fig. 3).

### Single use instrument

Problems: short timing life; smaller safety for patient; more difficulty of management (price, stocking and waste disposal); worse services (precision of cut, electronic support).

### Improvements

**Figure 4:**
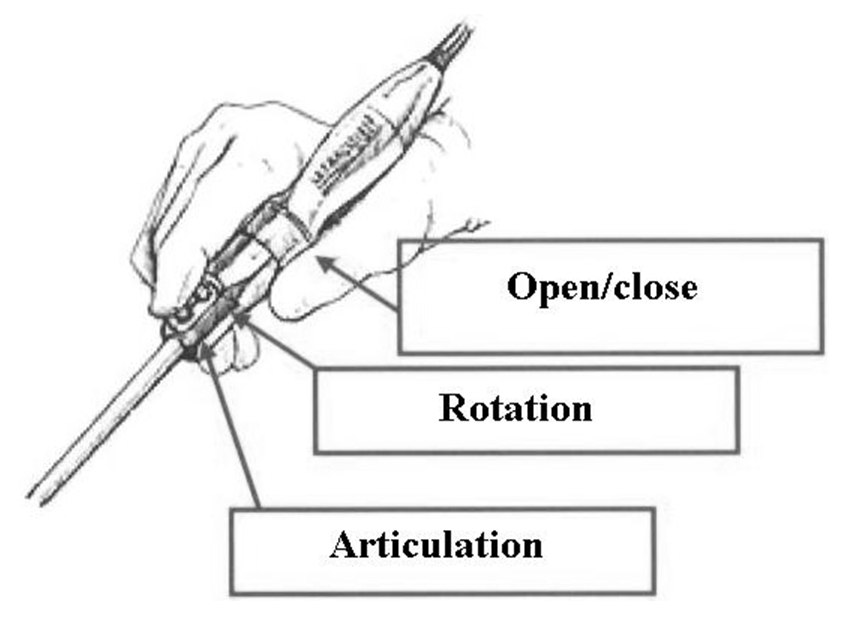
**Proposal for an electronic handle for the Roticulator Endo Mini-Shears™**.

Use of sterilised handle, in which electronic controls can be located, and single use shaft connecting to handle (fig. 4).

### Electric conduction

Breaking of insulating layer on the shaft; formation of conductive water layer on shaft.

### Improvements

**Figure 5:**
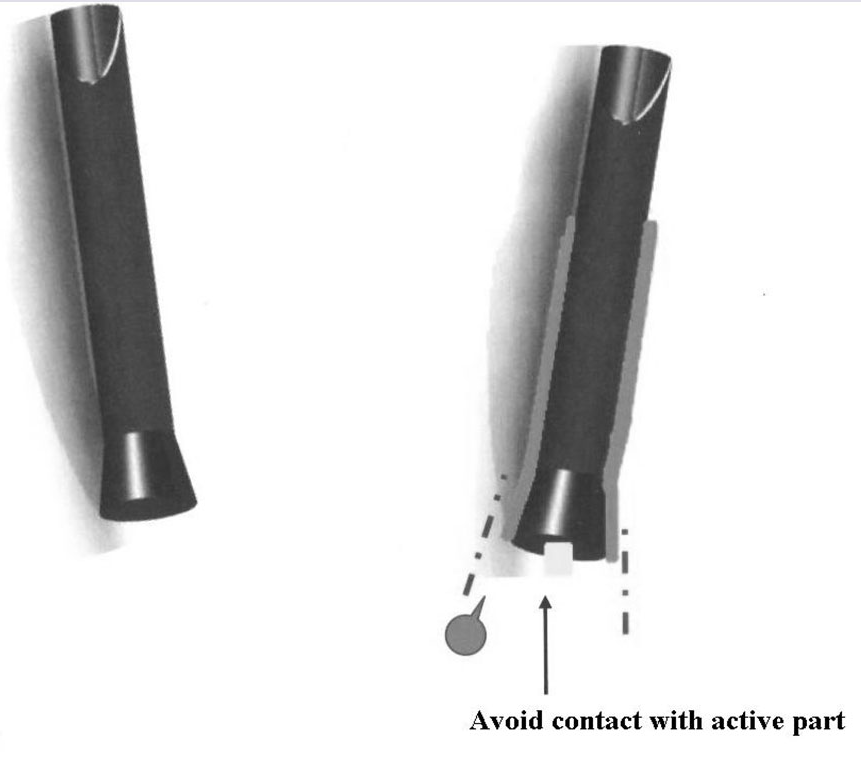
**Improvement proposal for the terminal portion of the Roticulator Endo Mini-Shears™**.

Replacement of the terminal part of insulating sheath with an insulating absorbent protective coating; replacement of the terminal linear shaft with a holding out coating, in order to avoid contact between active blade of scissors and shaft (fig. 5).

• Blunt tip trocar with balloon tip (Autosuture™, Norwalk, CT, USA)

The built-in adapters could break during their shifting on the entry port; the adapters aren't universal.

### Improvements

Replacement of the built-in adapters system with a more stable and versatile diaphragm-like system, controlled by an external gear.

• DST Series™ GIA™ 60 (Autosuture™, Norwalk, CT, USA)

1) The device is heavy, doesn't have an easy handling (first of all in the case of operators with small hands, like women), in particular when positioning the device on the tissue.

2) Firing clips needs too much strength.

### Improvements

**Figure 6:**
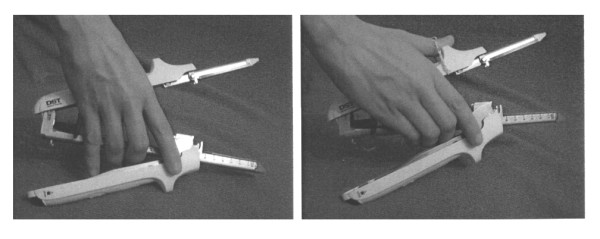
**Improvement proposal for the DST Series™ GIA™ 60**.

1) Addition of a ring on the external handle for the placement of the operating hand's thumb, in order to give a more correct and firmer handling of the device (fig. 6).

2) Reduction of friction and/or addition of a pneumatic firing system.

• EndoClinch™ II (Autosuture™, Norwalk, CT, USA)

1) The handle of the instrument is too tough, and a prolonged use may cause little pain to the surgeon's hands.

2) The drive that controls the ratchet is not very handy, either when turning on or turning off.

### Improvements

**Figure 7:**
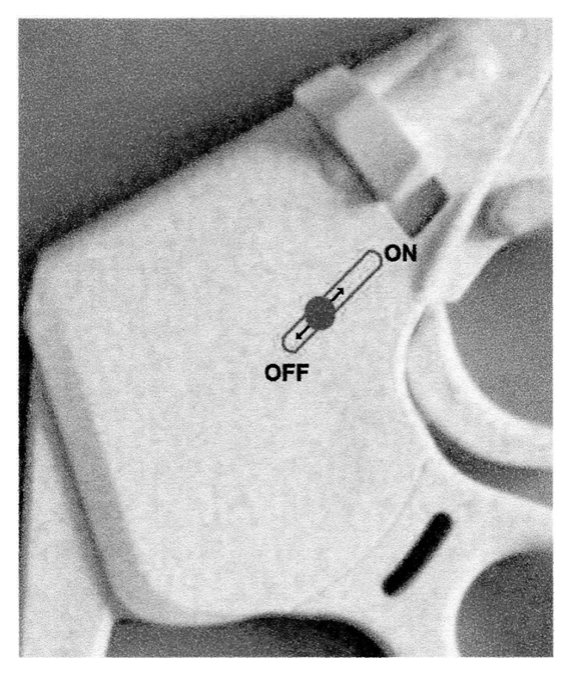
**Improvement proposal for the EndoClinch™ II**.

1) Application of small cushions on the handle itself, in order to give a more comfortable hold to the surgeon.

2) Placement of a sliding drive, similar to the drive that controls the ratchet, on the side of the instrument, near the gear (Fig. 7).

## Discussion

The advances in the experience with endoscopic video-assisted techniques have led to the introduction of dry laboratories in several centres. MIS requires training, crediting and case load, not always available in small centres. Therefore dry-lab training courses are necessary for improving the technical skills necessary to apply novel techniques [3-8]. Moreover, surgeons need to create an interface with engineers, in order to better understand the capabilities of a surgical device, enhance existent instrumentation, conceive new and smarter devices and eventually collaborate in the development of new ideas. On February this journal published papers from a panel of expert entitled "Innovation by surgeons" [9-20]. Some of these interesting contributions underline aspects concerning relationship between surgical academic research, technology transfer and business. Particularly, in their contribution, Heller, Michelassi and Schuler [14] give a fundamental contribution on the innovation process in the surgical field, outlining how doing translational research is only possible supporting effective communication between surgeons and biomedical engineers and promoting intercampus initiatives.

This kind of initiatives allows surgical department to devise a new academic mission: the technology transfer, in addition to teaching, research and clinical activities. This activity brings to production of intellectual property and eventual commercialization. In this field, the interface between surgeons and engineers leads naturally to innovation processes, which mean clinical results, fundraising and prestige for the academic institution.

A method has to be identified in order to develop a continuative collaboration: for example, having engineers participate to the operating room activities, analyzing needs, having a systematic approach to solutions, prototyping, running pre-clinical and clinical tests and possibly commercializing the product. Such an activity helps to overcome problems in the interdisciplinary research, letting brilliant ideas become real products.

For these reasons, we decided to test a group of medical engineering students in order to improve their practical and theoretical knowledge and their analysis skills about MIS instrumentation, and to analyze them while performing standardized MIS tasks on a pelvic trainer and eventually while presenting their essays about the devices they choose to analyze and criticize.

We used a simulator since it allows for practice at various levels [4]. Rosser et al [5] and Chung et al [6] were the first authors who devised standardized drills and measured laparoscopic skills using timing alone as an endpoint. In order to standardize our experience, we decided to assess trainees' skills with the same approach. The student's performance was evaluated through the five following tasks: 1) clipping and cutting; 2) cutting a paper; 3) positioning a needle; 4) intra-corporeal knotting; 5) carrying out specimen with EndoBag.

During this standardized hands-on experience, the students had the possibility to get comfortable with MIS devices. Such an experience is necessary in order to allow students to understand the advantages and shortcomings of their utilization during MIS procedures.

We had the possibility to appreciate how medical engineering students worked on the devices they utilized, producing significant improvement proposals about the devices they tested during the course, together with a significant improvement in performance with increasing skillness, as could be expected. The work in the dry lab seemed to highly stimulate a critical and innovative approach to every single technological devices.

In conclusion, we believe that devising a dry lab course for medical engineering students may be useful and innovative for teaching and improving analysis and management of laparoscopic devices, allowing identification of problems and *"brainstorming" *work during the hands-on experience, with the aim of developing better devices [21,22].

## Competing interests

The authors declare that they have no competing interests.

## Authors' contributions

FR: Designer and supervisor of the project. AM: Medical Engineer, tutor of the medical engineering students. GG: General Surgeon, tutor of the medical engineering students. GO: Responsible for statistical studies. MG: Responsible for statistical studies. AMF: Head of the Department of Surgery and responsible of the project. All authors read and approved the final manuscript.
